# Supplementary material for: Parvalbumin interneurons regulate rehabilitation-induced functional recovery after stroke and identify a rehabilitation drug
Source: Nat Commun. 2025 Mar 15;16:2556. doi: 10.1038/s41467-025-57860-0 (PMC11910580; doi:10.1038/s41467-025-57860-0)
Supplement: Supplementary file 1 — Supplementary Information [file 41467_2025_57860_MOESM1_ESM.pdf]

**Supplementary Figures**

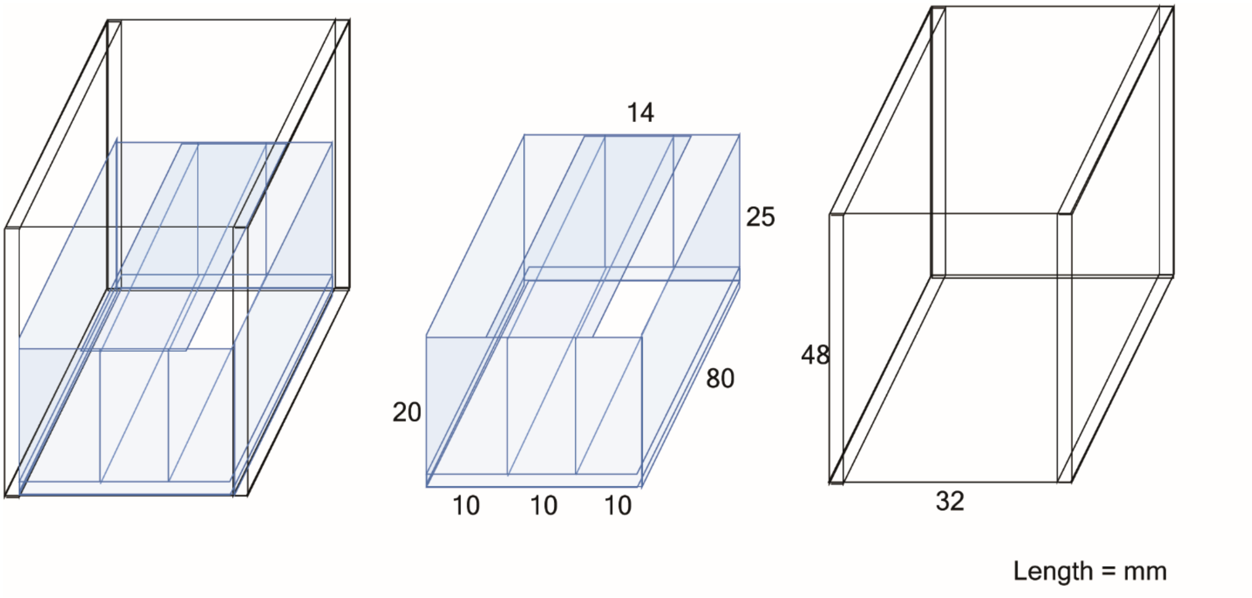


**Supplementary Fig. 1: Design of rehabilitation box.** The rehabilitation box consisted of an inner box with a center table and millet seed containers on either side and an outer box that contained the inner box. The narrow space between the center table and the ceiling of the outer box allows the mouse to crawl and reach down either side to pick up millet seeds but prohibits turning around. We confirmed that mice grab only one seed by a single reach. Therefore, the reach number exceeds the number of consumed millet seeds estimated from the calculation, the weight of consumed millet seeds divided by the average single millet seed weight. We also confirmed that mice use their tongue to retrieve the seeds if more than 4 g of seeds are provided but not with less amount.

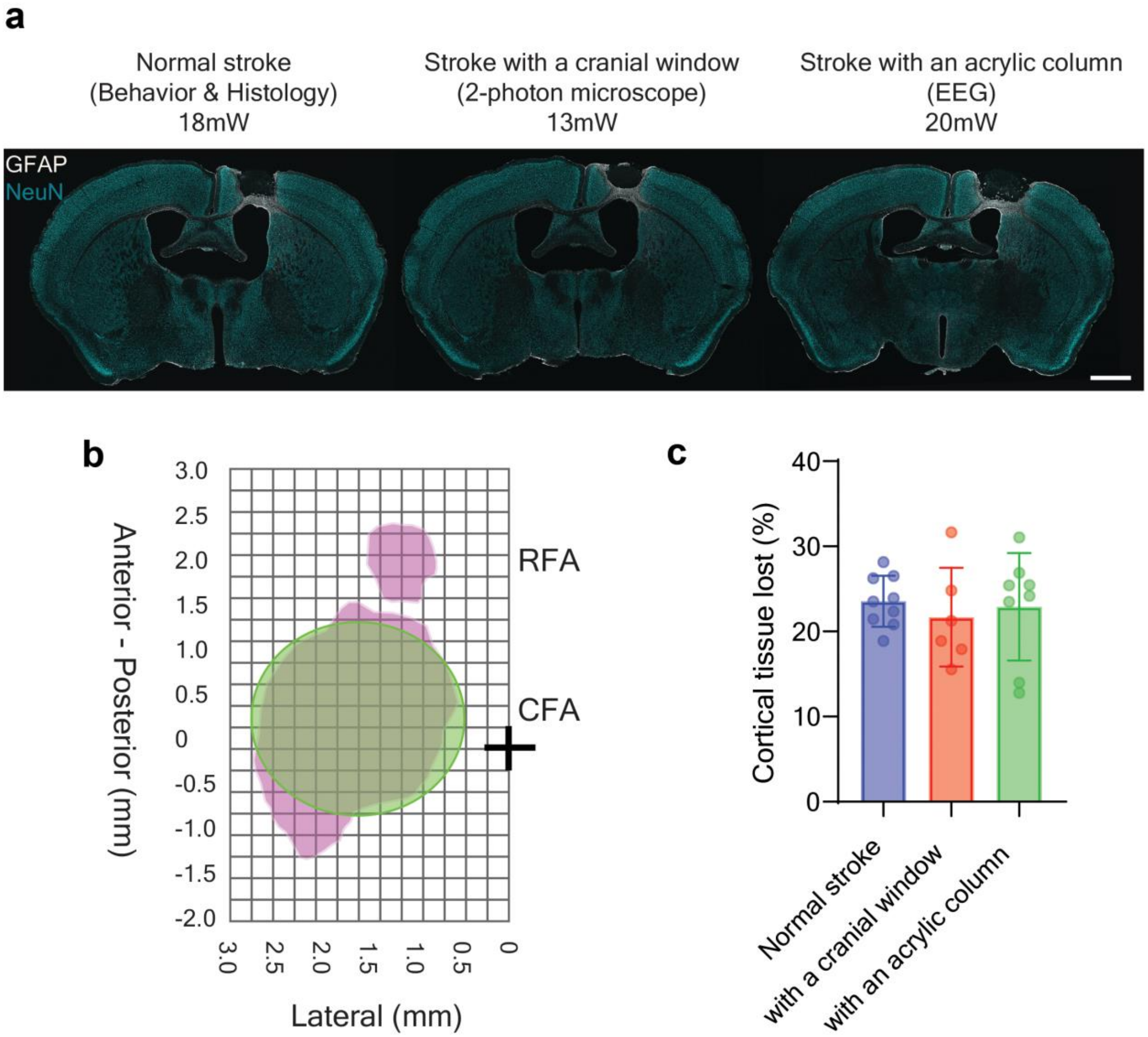


**Supplementary Fig. 2: Photothrombotic stroke models targeting caudal forelimb area. a**, representative images of brain slices with 3 types of photothrombotic stroke. Scale bar 1mm.**b**, The coordinate for laser illumination in the stroke models. The green circle indicates the area illuminated by a green laser. The pink area indicates the caudal forelimb area (CFA) in the motor cortex reported previously[110]. **c**, Cortical tissue damage by 3 stroke models.

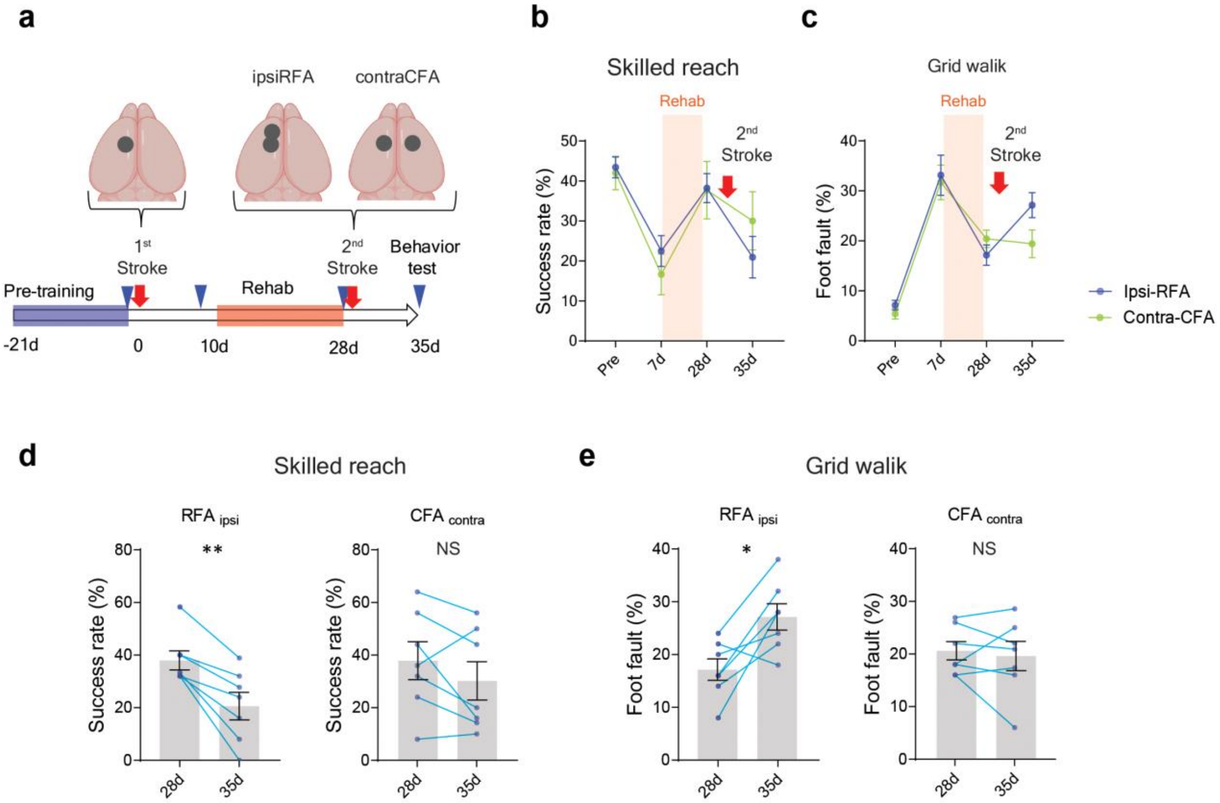


**Supplementary Fig. 3: Premotor cortex is a site of rehabilitation-induced recovery. a**, Timeline for the second stroke study. **b**,**c**, Motor performance in the skilled reach test (**b**) and the grid walk test (**c**). n = 7. **d**,**e**, Motor performance changes by the second stroke. Two-tailed paired t-test. *$P < 0.05$, **$P < 0.01$. (**a**) Created in BioRender. Carmichael, S. (2025) https://BioRender.com/l31s906.

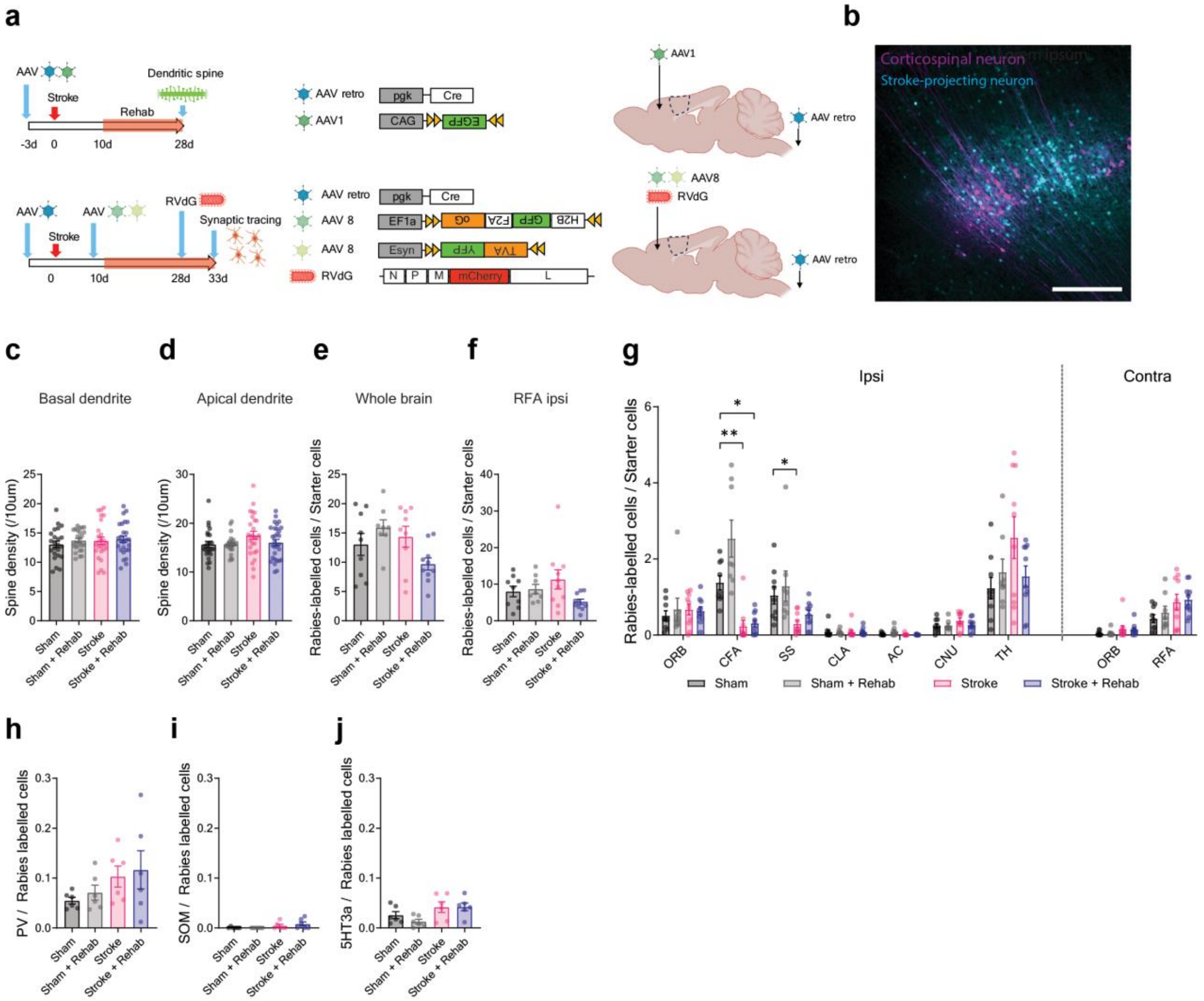


**Supplementary Fig. 4: Rehabilitation does not affect synaptic inputs to corticospinal neurons.** **a**, Timeline, virus vectors, and virus injection locations in dendritic spine analysis (upper) and monosynaptic tracing (lower). **b**, Representative image of corticospinal (retroAAV-CAG-tdTomato) and stroke-projecting neurons (retroAAV-CAG-GFP) showing rare overlap in these neuron types. c Scale bar 200 µm. **c**,**d**, Spine density in basal, n = 23 (Sham), 25 (Sham + Rehab), 26 (Stroke) or 26 (Stroke + Rehab) (**c**) and apical, n = 24 (Sham), 25 (Sham + Rehab), 27 (Stroke) or 30 (Stroke + Rehab) (**d**) dendrite. **e-g**, The number of RVdG-labelled cells normalized by the starter cells in the whole brain (**e**), RVdG injection neighbor: RFA (**f**), and distant brain areas (**g**). ORB: orbital area, CFA: caudal forelimb area, SS: somatosensory area, CLA: claustrum, AC: anterior cingulate area, CNU: cerebrum nuclei (striatum and pallidum), TH: thalamus. Kruskal-Wallis test. n = 9 (Sham), 8 (Sham + Rehab), 10 (Stroke) or 10 (Stroke + Rehab). **h-j**, The ratio of PV (**h**), SOM (**i**), 5HT3a (**j** in total local inputs. Kruskal-Wallis test. n = 6. *P < 0.05, **P < 0.01. (**a**) Created in BioRender. Carmichael, S. (2025) https://BioRender.com/l31s906.

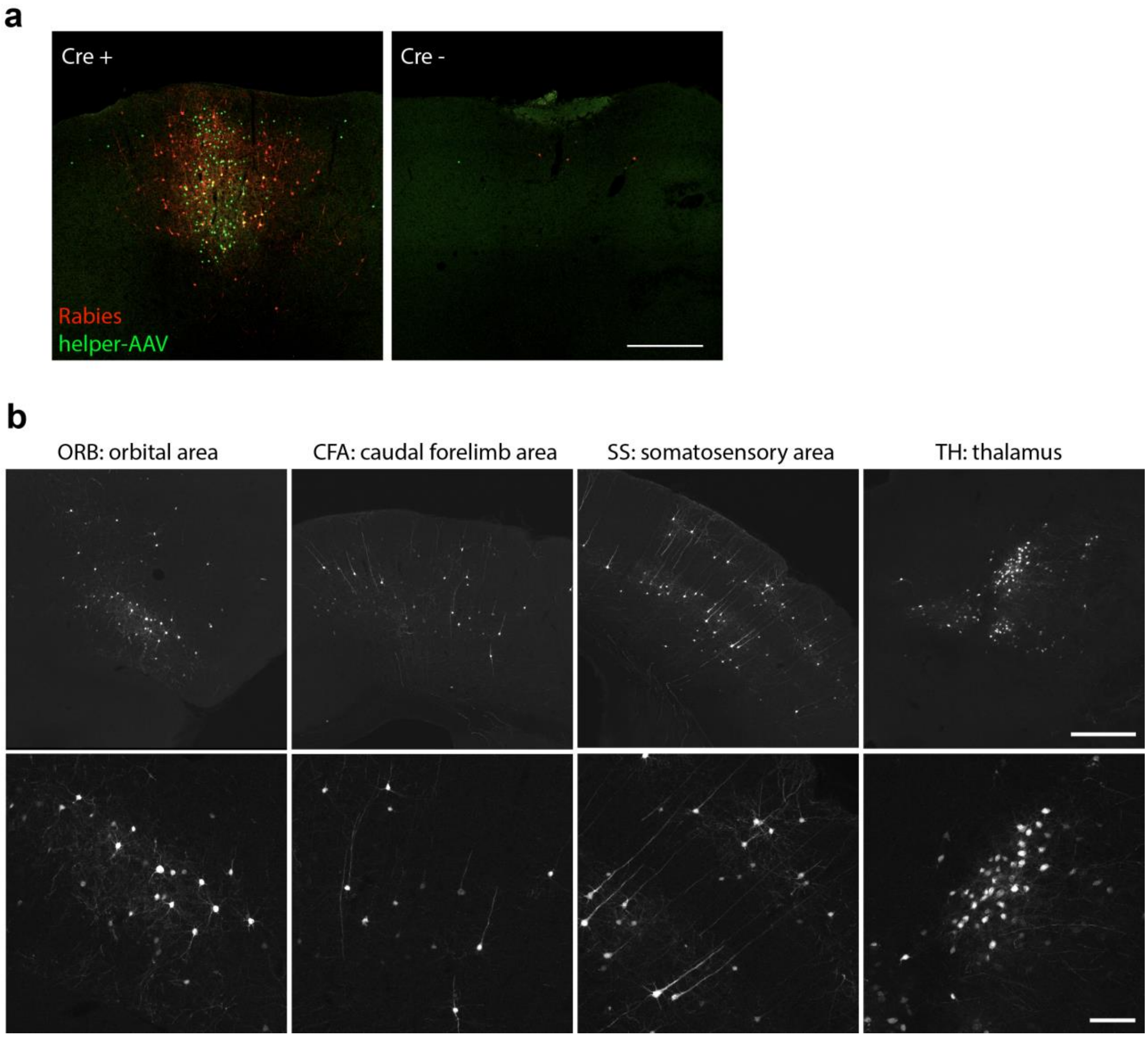


**Supplementary Fig. 5: Validation of rabies virus tracing. a**, Representative images of rabies virus and helper-AAV labeled neurons with or without Cre expression. Scale bar 200 µm. **b**, Representative images of rabies virus labeled neurons in the orbital area (ORB), caudal forelimb area (CFA), somatosensory area (SS), and thalamus (TH). Scale bar upper: 400 µm, lower 100 µm.

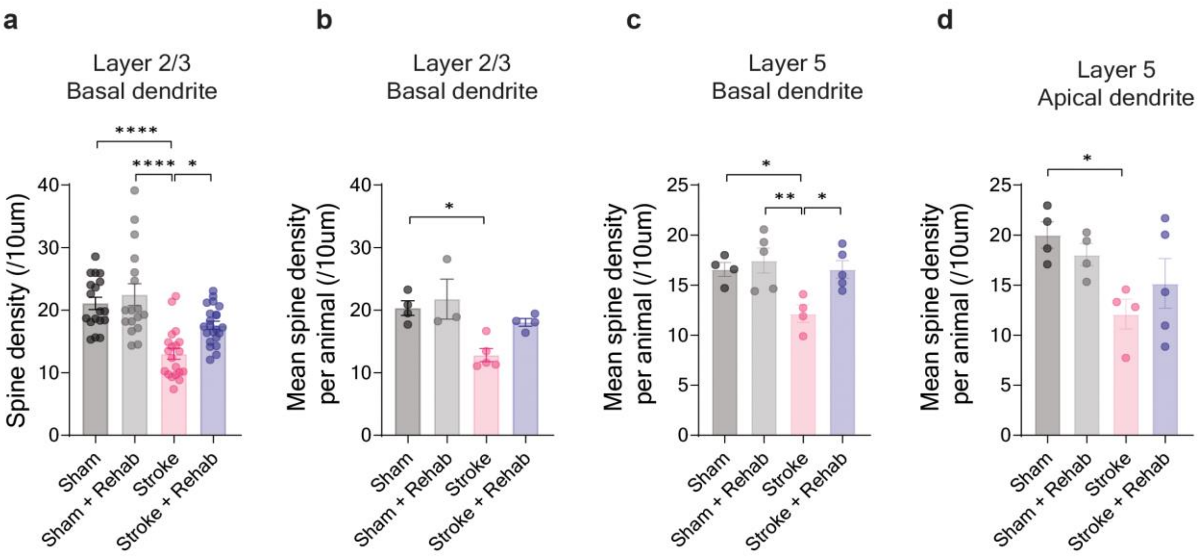


**Supplementary Fig. 6: Spine density in layer 2/3 stroke-projecting neurons and mean spine density in each animal. a**, Spine density in layer 2/3 stroke-projecting neurons, n = 19 (Sham), 17 (Sham + Rehab), 20 (Stroke) or 22 (Stroke + Rehab). **b-d**, Average spine density of layer 2/3 basal (**b**), layer 5 basal (**c**), and apical dendrites (**d**). Spine densities were averaged in each animal. n = 3-5 / group. Kruskal-Wallis test. *P < 0.05, **P < 0.01, **** P < 0.0001.

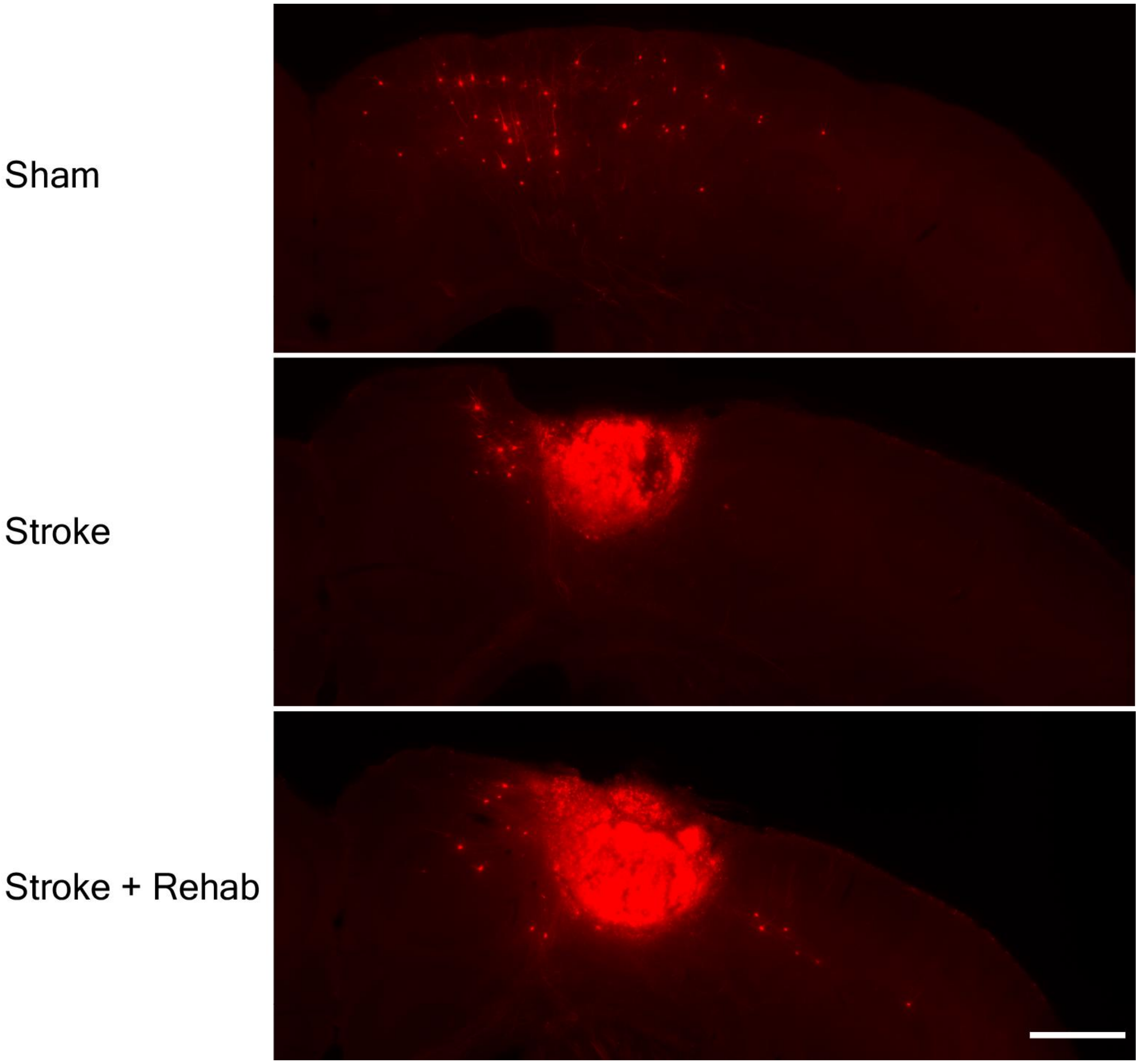


**Supplementary Fig. 7: Synaptic connection from peri-infarct tissue to stroke-projecting neurons.** Representative images of peri-infarct cortical tissue with rabies virus labeling. Scale bar 500 µm.

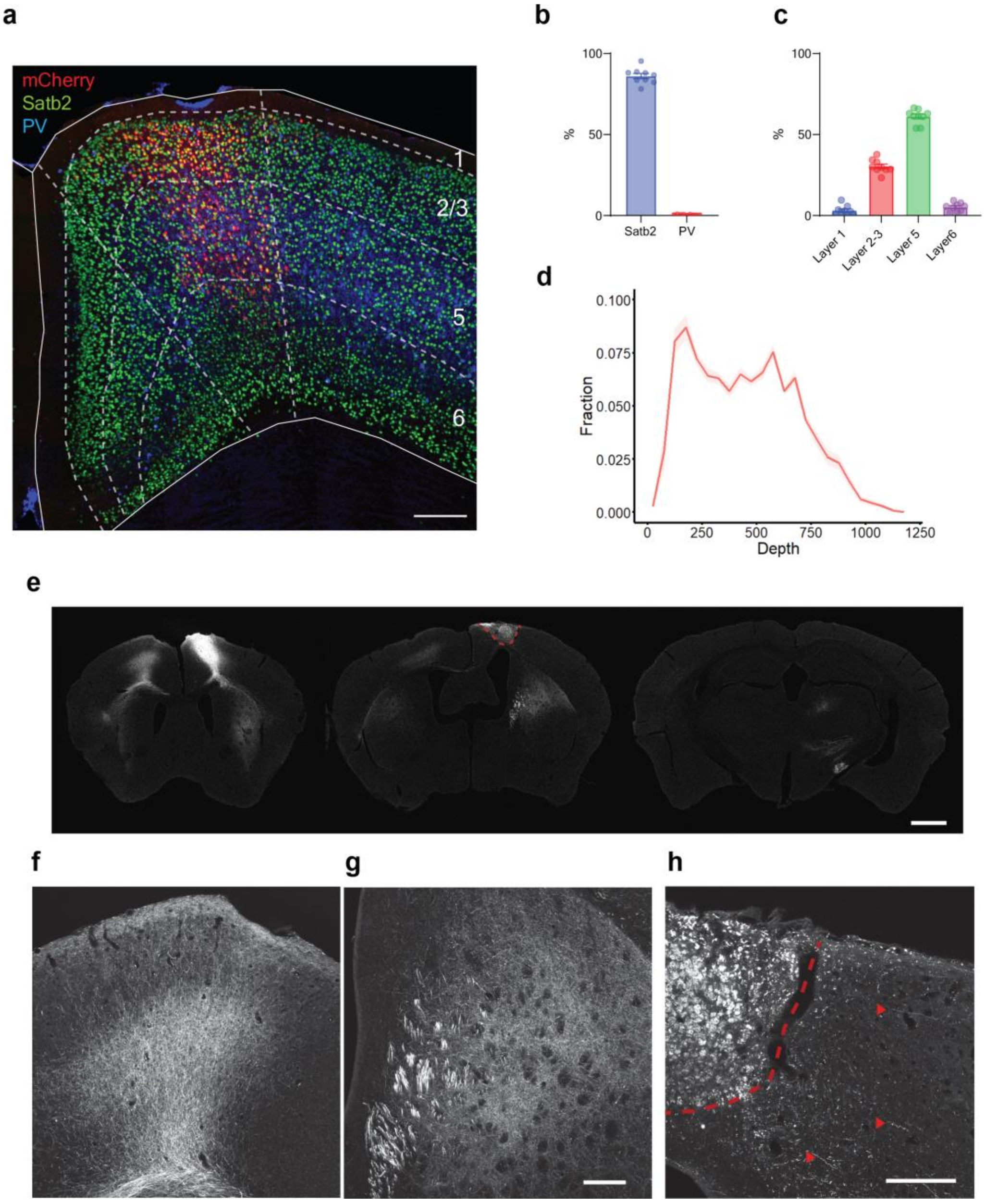


**Supplementary Fig. 8: Layer distribution and axon projection of stroke-projecting neurons. a**, Representative image of stroke-projecting neurons. Scale bar 200 μm. **b**, Proportion of Satb2 and PV expression in stroke-projecting neurons. **c**, Proportion of stroke-projecting neurons in each cortical layer. **d**, Layer distribution of stroke-projecting neurons by depth. **e-h**, Representative pictures of axonal projections from stroke projecting neurons. Dense axon projections exist in the contralateral cortex (**e**-left,**f**), striatum (**e**-center,**g**), thalamus (**e**-right), and cerebral peduncle (**e**-right), but the peri-infarct projection was sparse (**e**-left,**h**). The Red dashed line indicates the infarct border, and the red triangles indicate axons. Scale bar 1.0 mm (**e**) and 200 μm (**f-h**).

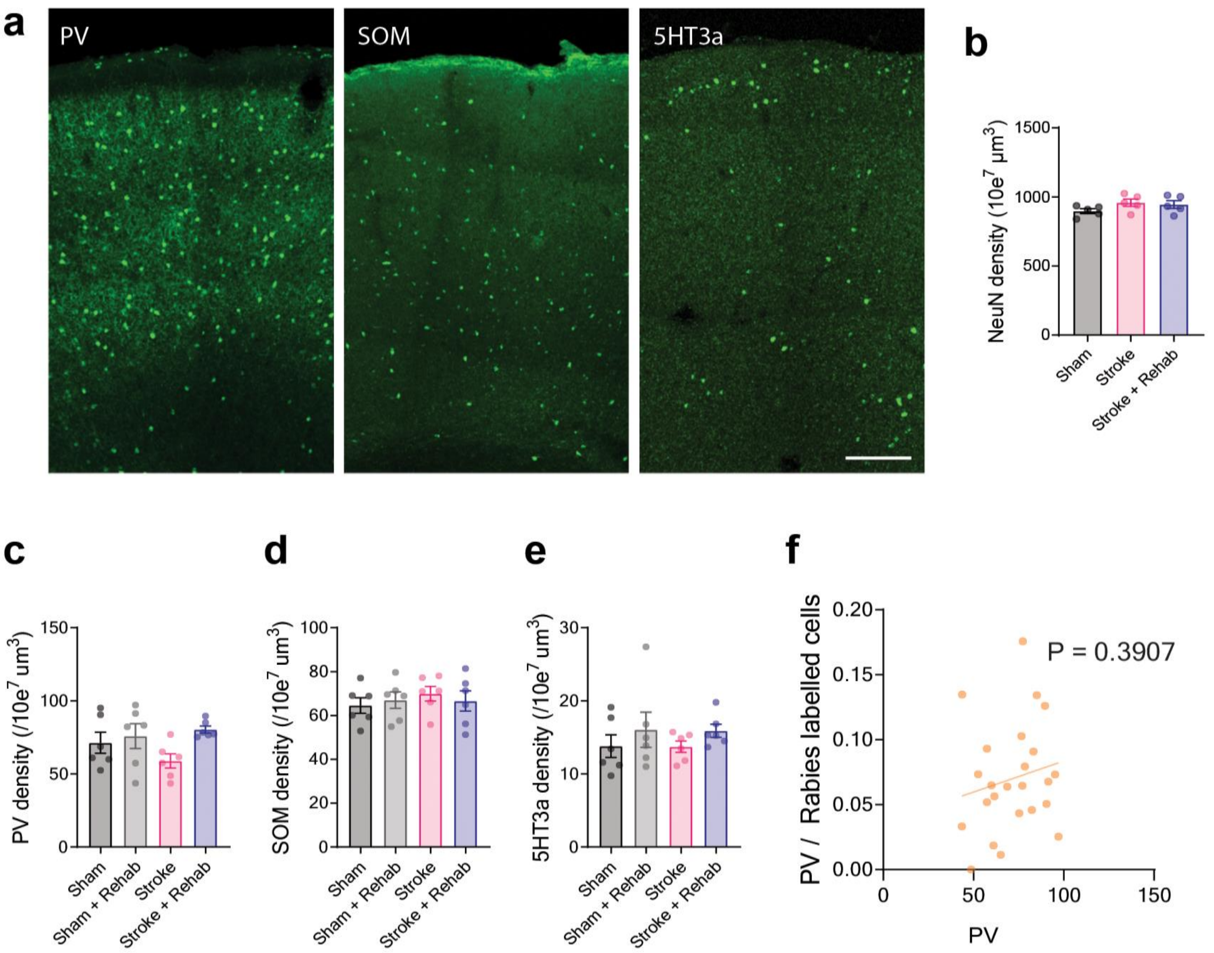


**Supplementary Fig. 9: Stroke does not change the density of interneurons. a**, Representative images of PV, SOM and 5HT3a interneurons in premotor cortex. Scale bar 200 μm. b, Density of NeuN positive neurons. n = 5. **c**-**e**, Density of PV (**b**), SOM (**c**), and 5HT3a interneurons (**d**). n = 6. **f**, Correlation between the PV interneuron density and the ratio of PV labelled stroke-projecting neurons. Pearson correlation (n = 24, r = 0.183, P=0.3907).

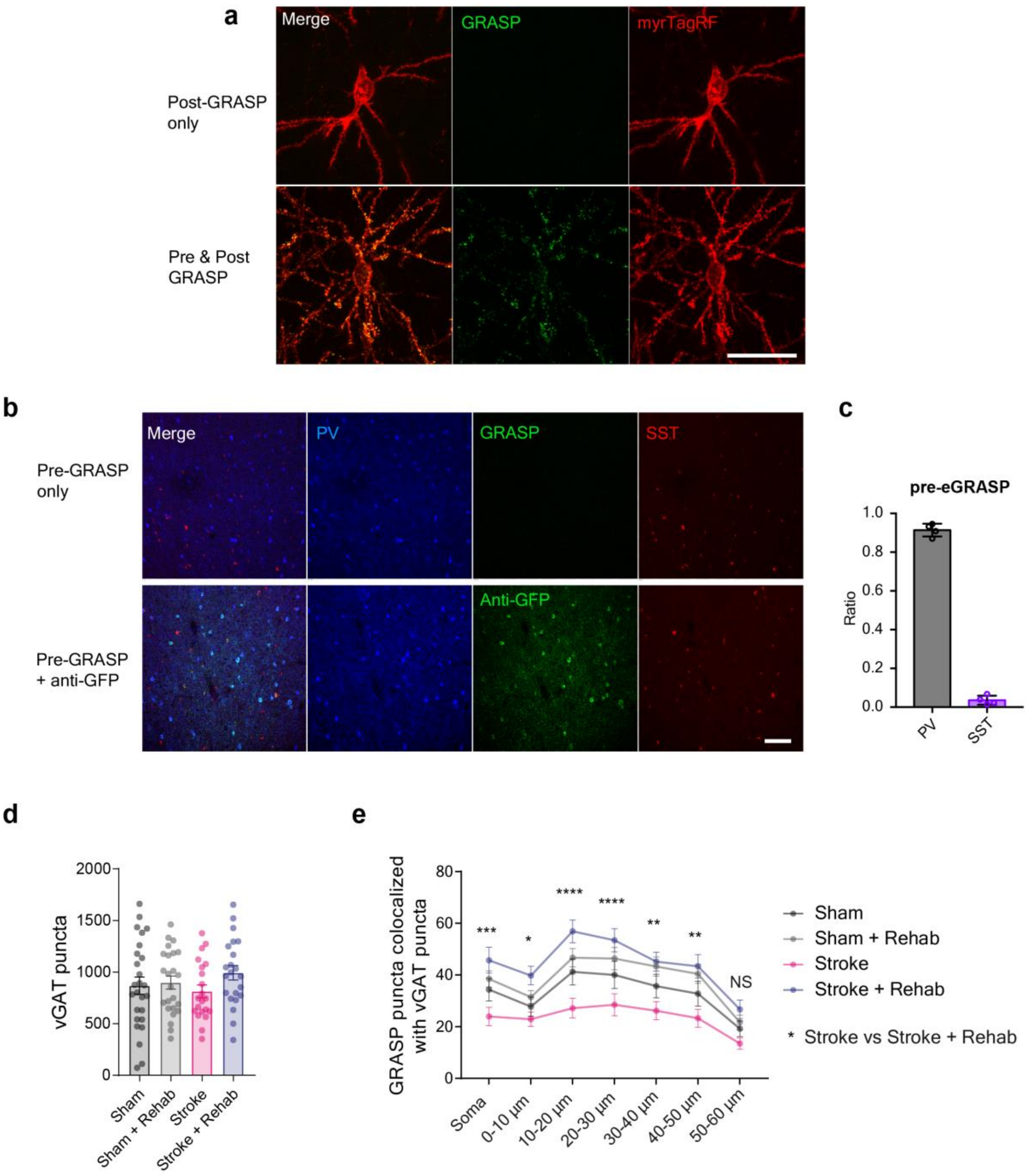


**Supplementary Fig. 10: Protocol validation and additional analysis in GRASP study. a**, Representative images of GRASP with or without Pre-GRASP expression. Scale bar 50 μm. **b**, Representative images of GRASP without Post-GRASP. Scale bar 100 μm. **c**, PV interneuron specificity of AAV-S5E2 vector. **d**, The number of  vGAT positive synapses. Kruskal-Wallis test. n = 24 (Sham), 24 (Sham + Rehab), 21 (Stroke) or 22 (Stroke + Rehab). *P < 0.05, **P < 0.01. **e**. Spatial distribution of GRASP puncta. Two-way ANOVA, F (3, 609) = 33.55, P < 0.0001, Tukey's multiple comparison test (Stroke vs Stroke + Rehab). *P < 0.05, **P < 0.01, ***P < 0.001, ****P < 0.0001.

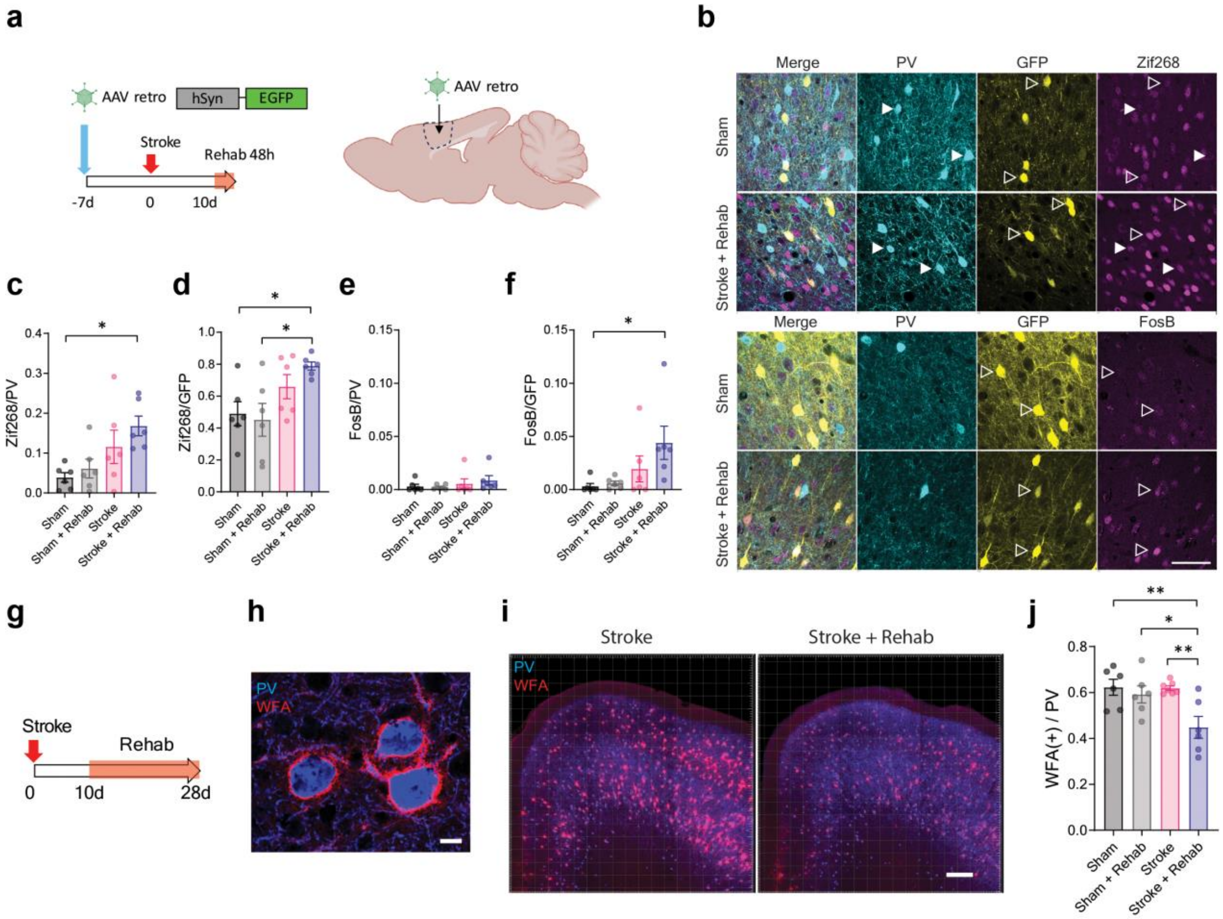


**Supplementary Fig. 11: Rehabilitation induces PV interneuron activation and plasticity. a**, Timeline, virus vector, and injection site for the immediate early gene study. **b**, Representative image of PV interneurons and stroke-projecting neurons (GFP labelled) with immediate early gene, Zif268 and FosB. Scale bar 50 µm. The closed and open triangles indicate the locations of the PV interneurons and the stroke-projecting neurons. **c**,**d**, Ratio of Zif268 positive PV interneurons (**c**, F (3, 20) = 4.388, P=0.0158) and stroke-projecting neurons (**d**, F (3, 20) = 4.309, P=0.0169). One-way ANOVA, Tukey's multiple comparisons test. n =6. **e**,**f**, Ratio of FosB positive PV interneurons (**e**) and stroke-projecting neurons (**f**). Kruskal-Wallis test, *P < 0.05. **g**, Timeline for the perineuronal nets study. **h**, Representative image of perineuronal nets stained with Wisteria floribunda agglutinin (WFA). Scale bar 10 µm. **i**, Representative images of perineuronal nets in premotor cortex in stroke animals with or without rehabilitation. Scale bar 300 µm. **j**, Ratio of the PV interneurons surrounded by perineuronal nets. One-way ANOVA, F (3, 21) = 5.811, P=0.0047, Tukey's multiple comparisons test. n = 6. *P < 0.05, **P < 0.01. (**a**) Created in BioRender. Carmichael, S. (2025) https://BioRender.com/l31s906.

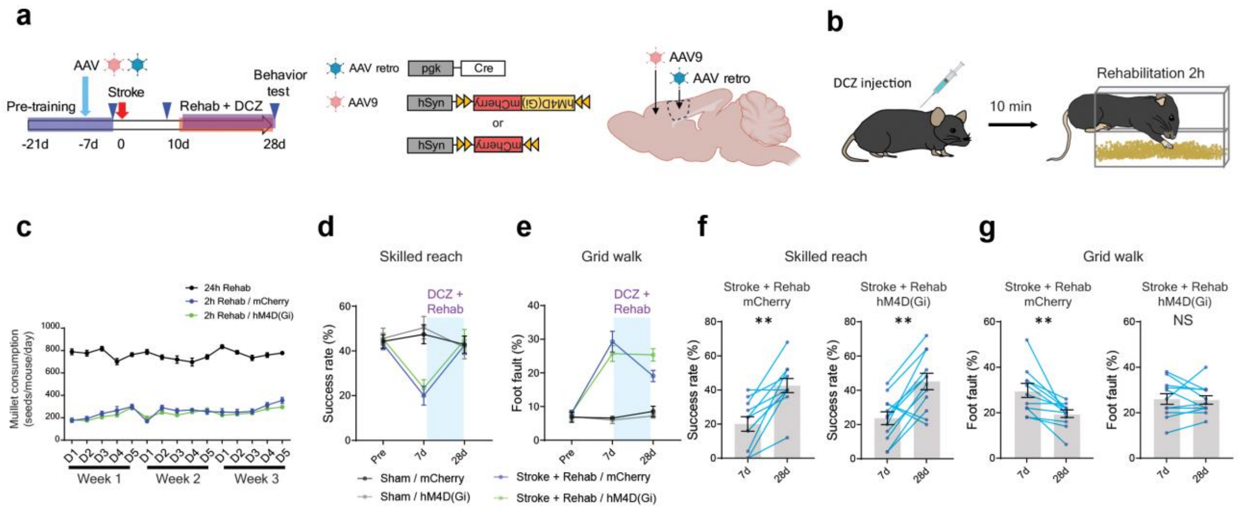


**Supplementary Fig. 12: Activation of stroke projecting neurons is necessary for recovery of grid walk but not for skilled reaching behavior.** **a**, Timeline, virus vectors, and injection sites in the chronic chemogenetic inhibition targeting stroke-projecting neurons. **b**, Procedure for rehabilitation with chemogenetic inhibition. **c**, Millet seed consumption with 2-hour rehabilitation. n = 6. **d**,**e**, Motor performance in the skilled reaching test (**d**, time by group, F (6, 72) = 8.406, P<0.0001) and the grid walk test (**e**, time by group, F (6, 72) = 15.22, P<0.0001). n = 7 (Sham/mCherry), 10 (Sham/hM4D), 11 (Stroke+Rehab/mCherry) or 12 (Stroke+Rehab/hM4D). **f**,**g**, Functional recovery by rehabilitation in the skilled reaching test (**f**) and the grid walk test (**g**). Two-tailed paired t-test. **P < 0.01. (**a,b**) Created in BioRender. Carmichael, S. (2025) https://BioRender.com/l31s906.

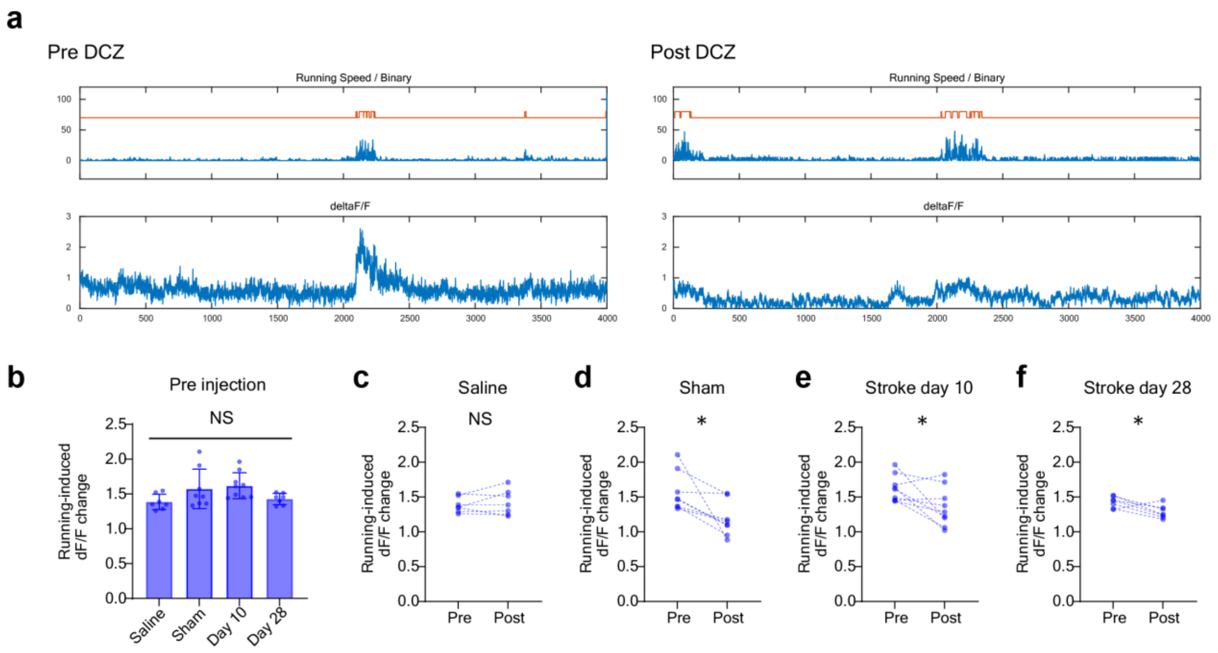


**Supplementary Fig. 13: DREADD inhibition of PV interneurons. a**, Representative motion and delta F/F traces before and after DCZ injection. **b**, Mean running-induced delta F/F change before DCZ injection. One-way ANOVA, F (3, 27) = 2.754. P = 0.0619. **c-f**, Mean running-induced delta F/F change before and after DCZ injection. Two-tailed paired-t test. n = 7 (Saline), 8 (Sham), 9 (Stroke day 10), or 7 (Stroke day 28). *P < 0.05.

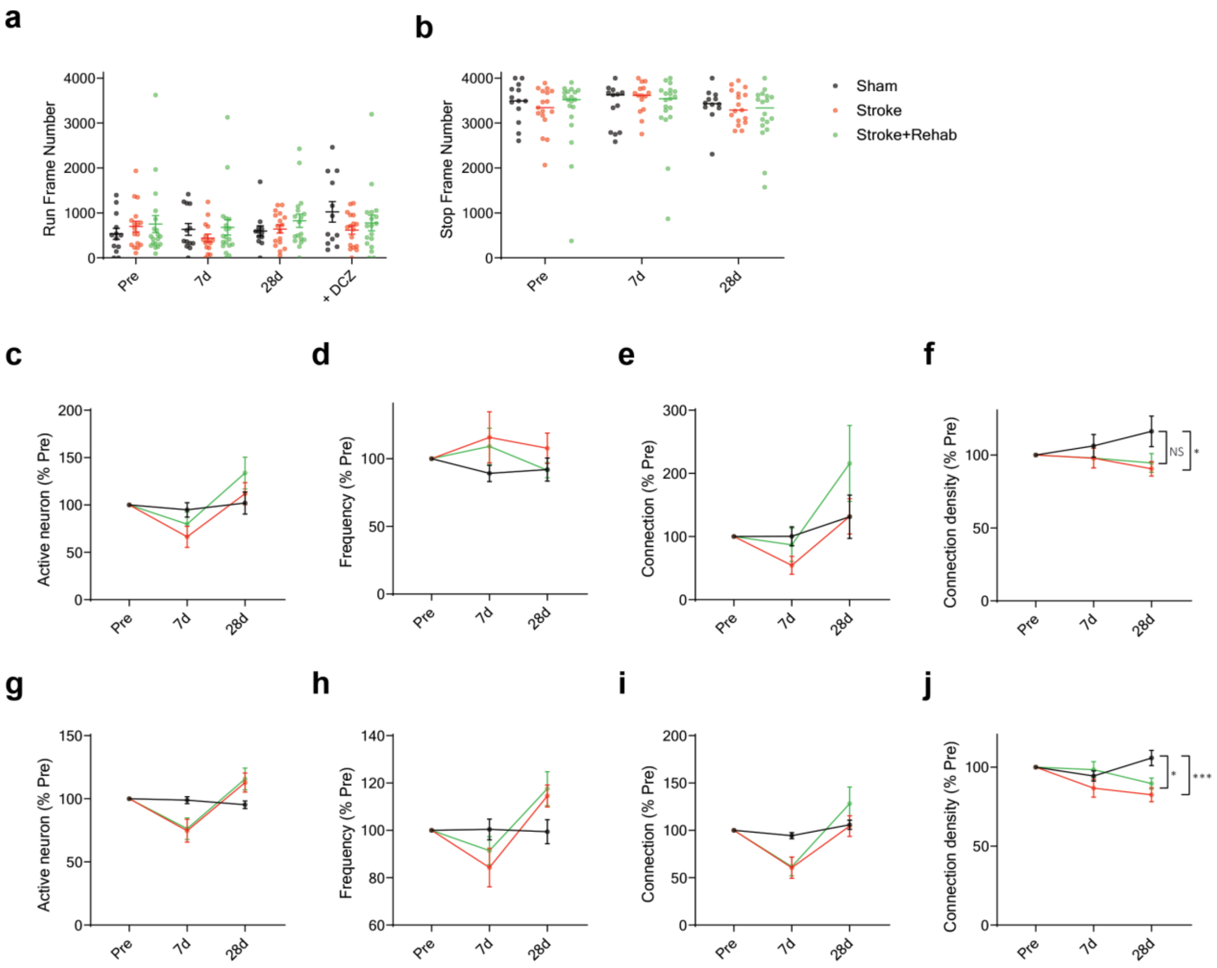


**Supplementary Fig. 14: Neuronal activity changes in voluntary running after stroke. a**,**b**, Frame number of the run (**a**) and stop (**b**) epoch. n = 13 (Sham), 17 (Stroke) or 19 (Stroke + Rehab) **c-j**, Neuronal activity changes in the run (**c-f**) and stop (**g-j**) epochs. Stroke causes a slight decrease in the active neuron (**g**) and the connection number (**i**) in the stop epoch. Like forced running, stroke caused a persistent decrease in connection density, emerging with delay (**f**,**j**). Mixed-effects model, Sidak’s multiple comparisons test. *P < 0.05, ***P < 0.001.

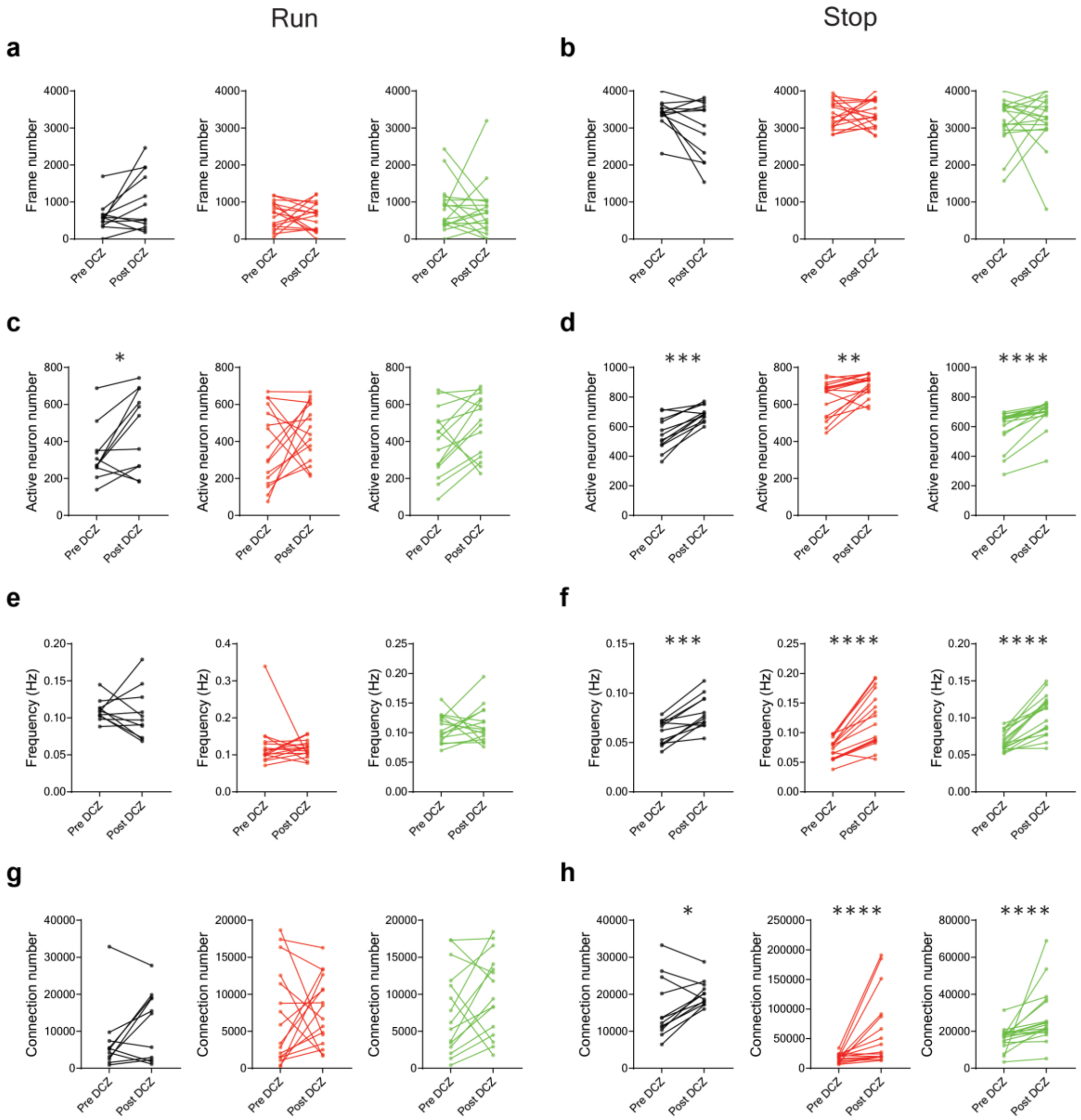


**Supplementary Fig. 15: Neuronal activity changes induced by PV interneuron inhibition in healthy and stroke animals. a**,**b**, Frame number changes by DCZ injection in the run (**a**) and stop epochs (**b**). n = 13 (Sham), 17 (Stroke) or 19 (Stroke + Rehab). **c-h**, Changes in the active neuron (**c**,**d**), calcium transient frequency (**e**,**f**), and connection number (**g**,**h**) in the run (**c,e,g**) and the stop epochs (d,f,**h**). Two-tailed Wilcoxon test. *P < 0.05, **P < 0.01, ***P < 0.001, ****P < 0.0001.

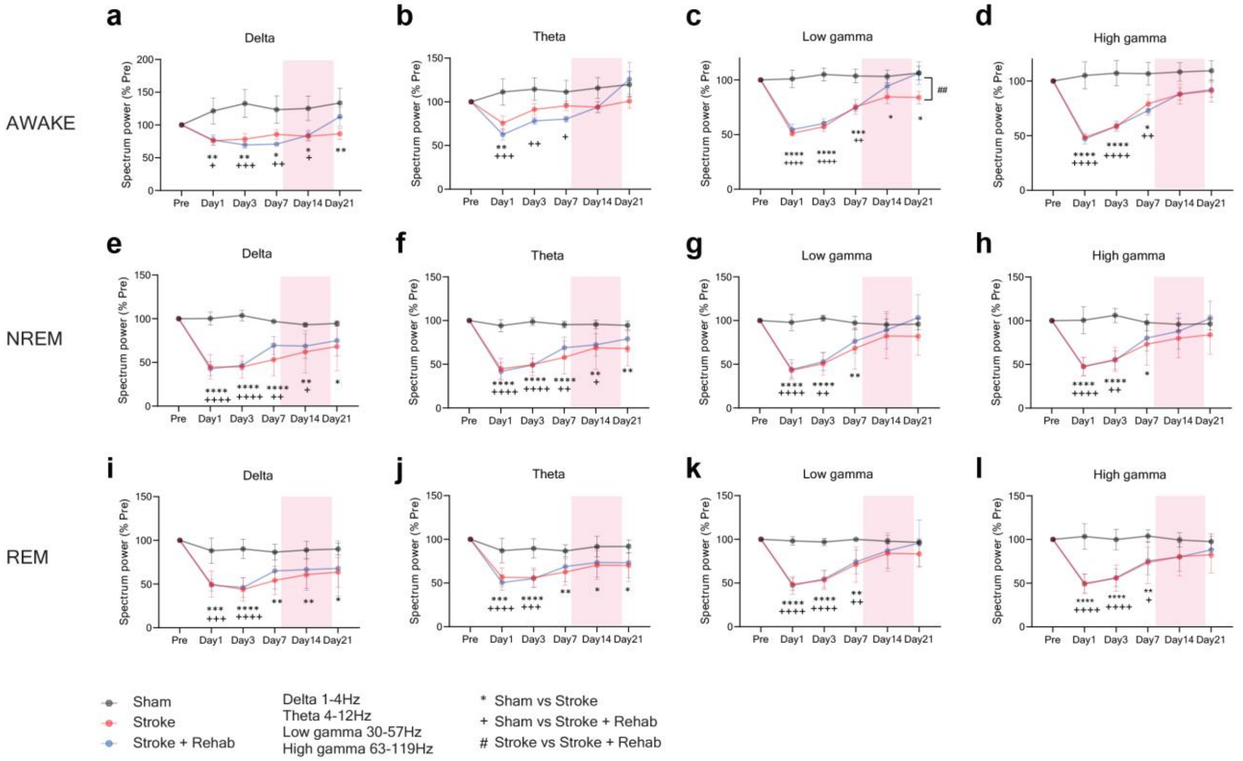


**Supplementary Fig. 16: Spectrum power changes after stroke in three vigilant states.** Spectrum power in AWAKE (**a-h**), NREM (**e-h**), and REM periods (**i-l**). Red rectangles indicate the rehabilitation period. Stroke-induced global spectral power decrease ranging delta (**a**,**e**,**i**). theta (**b**,**f**,**j**), low gamma (**c**,**g**,**k**), and high gamma frequency bands (**d**,**h**,**l**). The spectrum powers remained significantly reduced in delta and low gamma frequency during the AWAKE period. In contrast, the spectrum powers remained reduced in delta and theta frequency during the NREM and REM periods. Mixed-effects model, *P <0.05, **P <0.01, ***P <0.001, ****P < 0.0001: Sham vs Stroke, + P < 0.05, ++ P < 0.01, +++ P < 0.001, ++++ P < 0.0001: Sham vs Stroke + Rehab, ## P < 0.01: Stroke vs Stroke + Rehab, Tukey's multiple comparisons test. n = 4 (Sham), 7 (Stroke) or 5 (Stroke+Rehab).

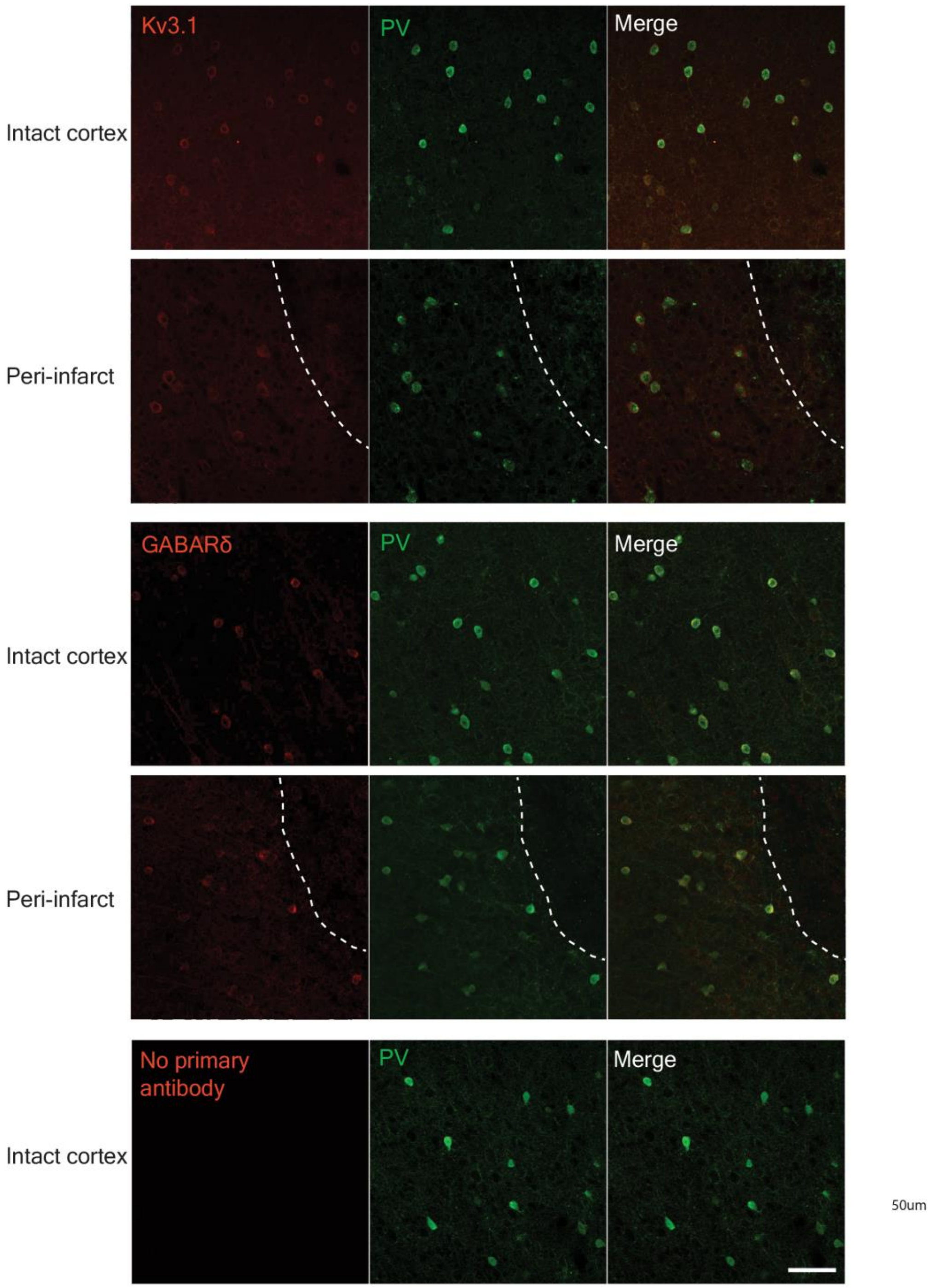


**Supplementary Fig. 17: Kv3.1 and GABAARδ expression in intact and peri-infarct cortex.** Kv3.1 and GABAARδ detected by specific antibodies colocalized with PV in both intact and peri-infarct cortex, while no primary antibody staining makes no signal. Scale bar 50 μm.

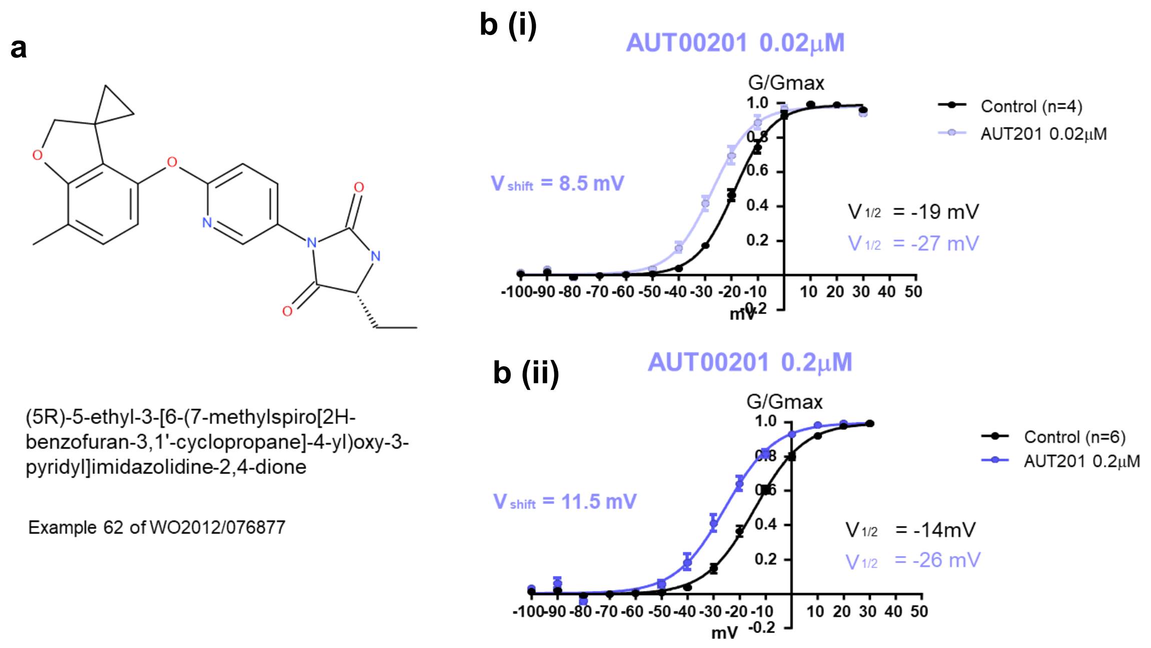


**Supplementary Fig. 18: In vitro activity of AUT00201 determined by whole-cell patch clamp electrophysiology.** AUT00201 shifts the voltage dependent activation of Kv3.1 channels to more negative potentials. (**a**) The structure and full UPAC chemical name of AUT00201 (**b**) Effects of AUT00201 on the activation voltage-dependence of Kv3.1 channels were evaluated by plotting normalized conductance as a function of membrane voltages

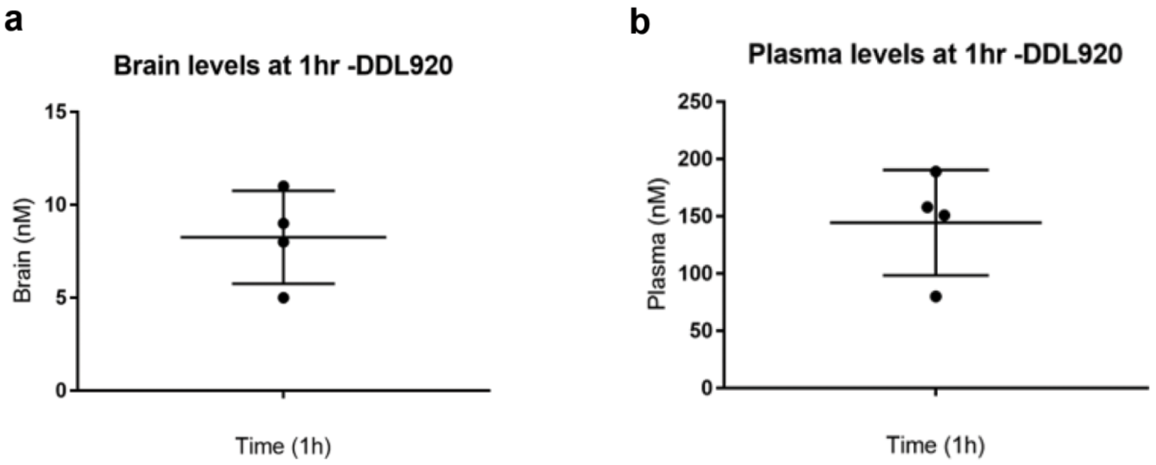


**Supplementary Fig. 19: DDL-920 pharmacokinetics.** (a) Average DDL-920 brain levels; 1 hour post-administration via pipetted feeding. (**b**) Average DDL-920 plasma levels; 1 hour post-administration via pipetted feeding

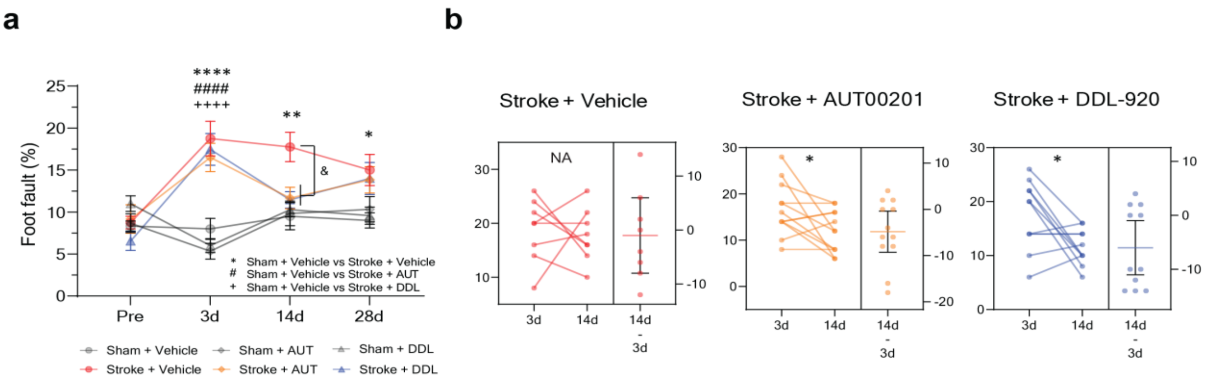


**Supplementary Fig. 20: Effects of the PV interneuron-activating drugs in the grid walk test. a,** Foot faults in the grid walk test. Two-way repeated measure ANOVA, *P < 0.05, **P < 0.01, ****P < 0.0001: Sham + vehicle vs Stroke + Vehicle, ####P < 0.0001: Sham + vehicle vs Stroke + AUT, ++++P < 0.0001: Sham + vehicle vs Stroke + DDL, &P < 0.05: Stroke + vehicle vs Stroke + AUT (P = 0.0404) / Stroke + DDL (P = 0.0351), Sidak's multiple comparison test. n = 12 (Sham+Vehicle), 12 (Sham+AUT), 14 (Sham+DDL), 8 (Stroke+Vehicle), 12 (Stroke+AUT), 11 (Stroke+DDL). **b**, Functional recovery from day 3 to day 14. Two-tailed paired t-test, day 3 vs day 14. *P < 0.05.

**Supplementary tables.**

## A. Patient characteristics

| | |
|---|---|
| N | 27 |
| Side of infarct | R16/L11 |
| Infarct volume (median, IQR) | 4.8 cc [1.4 – 37.0] |
| Age (years) | 58 [49 – 67] |
| Time from stroke to Visit 1 (days) | 12 [8 - 17] |
| Stroke type | Ischemic 21/intracerebral hemorrhage 6 |
| Gender | 20M/7F |

## B. Low gamma V1

| | **Healthy control** | **Patients with stroke** | **p** |
|---|---|---|---|
| V1 low gamma power cM1 | 0.037 [0.028 - 0.057] | 0.026 [0.02 - 0.052] | 0.18 |
| V1 low gamma power iM1 | 0.043 [0.027 - 0.058] | 0.038 [0.019 - 0.057] | 0.49 |
| V1 low gamma power cPMd | 0.041 [0.028 - 0.061] | 0.035 [0.019 - 0.085] | 0.59 |
| V1 low gamma power iPMd | 0.042 [0.03 - 0.062] | 0.053 [0.028 - 0.079] | 0.71 |
| V1 low gamma power SMA | 0.028 [0.021 - 0.045] | 0.031 [0.016 - 0.046] | 0.7 |

## C. Low gamma change V1-V5

| **Brain region** | **V5-V1 change in low gamma power** | **P** | **V5-V1 % change in low gamma power** | **P** |
|---|---|---|---|---|
| cM1 | 0.023 [-0.004 – 0.062] | 0.05 | 91.9 [-12.4 - 180] | 0.008 |
| iM1 | 0.015 [-0.003 – 0.044] | 0.045 | 46.4 [-6.0 – 170] | 0.008 |
| cPMd | 0.015 [-0.028 – 0.05] | 0.22 | 37.0 [-37.3 – 206.3] | 0.059 |
| iPMd | 0.014 [-0.001 – 0.032] | 0.13 | 26.9 [-2.3 – 69.4] | 0.045 |
| SMA | -0.002 [-0.01 – 0.028] | 0.33 | -13.6 [-28.0 – 212.6] | 0.17 |

**Supplementary table 1: Information on the human EEG study.**

**A**. Patient characteristics. **B**. Low gamma power at Visit 1. Two-tailed t-test. **C**. Low gamma change from V1 to V5. Wilcoxon signed-rank test. Data are presented as median and IQR.

| AAV (Packaging in the lab) | | | Titer | Experiment |
|---|---|---|---|---|
| Plasmid #135635 & #111598 | pAAV-S5E2-pre-eGRASP(p32) | PHPeB | 1.87x10E12 | GRASP |
| Plasmid #111581 | pAAV-EWB-DIO-myrTagRFP-T-P2A-post-eGRASP | PHPeB | 2.77x10E12 | GRASP |
| Plasmid #105558 & #167572 | pENN-AAV-CaMKII-Ribo-jGCaMP8s-WPRE | PHPeB | 4.62x10E13 | Calcium imaging |
| AAV (Addgene) | | | | |
| 50465-AAVrg | pAAV-hSyn-EGFP | AAVrg | 1.1x10E13 | Immediate early gene |
| 51502-AAV1 | AAV pCAG-FLEX-EGFP-WPRE | AAV1 | 1.0x10E13 | Dendritic spine |
| 24593-AAVrg | AAV-pgk-Cre | AAVrg | 9.3x10E12 | Stroke-projecting neuron |
| 44362 | pAAV-hSyn-DIO-hM4D(Gi)-mCherry | AAV9 | 2.3x10E13 | Chemogenetics |
| 50459 | pAAV-hSyn-DIO-mCherry | AAV9 | 2.1x10E13 | Chemogenetics |
| AAV (Salk institute) | | | | |
| 120269 | AAV1-Esyn-DIO-TVA-YFP | AAV1 | 1.13x10E12 | Monosynpatic tracing |
| 74289 | AAV8-FLEX-H2B-GFP-2A-oG | AAV8 | 4.57x10E12 | Monosynpatic tracing |
| RV (Salk institute) | | | | |
| 32635 | EnvA G-Deleted Rabies-mCherry | RV | 1.28x10E8 | Monosynpatic tracing |

| **antibody** | **company** | **cat#** | **Host** | **Dilution** |
|---|---|---|---|---|
| Parvalbumin | Abcam | ab11427 | Rabbit | 1:1000 |
| Parvalbumin | Swant | 1275 | mouse | 1:1000 |
| Parvalbumin | Synaptic system | 195 004 | Guinea Pig | 1:1000 |
| Somatostatin | Peninsula lab | T-4103 | Rabbit | 1:1000 |
| Satb2 | Abcam | ab92446 | Rabbit | 1:1000 |
| vGat | Synaptic system | 131 003 | rabbit | 1:1000 |
| FosB | CST | 2251 | rabbit | 1:1000 |
| EGR1 (Zif268) | CST | 4154 | rabbit | 1:1000 |
| Kv3.1 | Alomone | APC-014 | Rabbit | 1:500 |
| GABARdelta | Addgene | 180118-rAb.T | Mouse | 1:200 |
| NeuN | Abcam | ab177487 | Rabbit | 1:1000 |
| GFAP | Life technology | #130300 | rat | 1:500 |

| **HCR probe** | **company** |
|---|---|
| Htr3a | Molecular Instrument |

**Supplementary table 2: Virus vectors and antibodies**

| Figure | | Experiment | Strain | Number of animals | | | | Note |
|---|---|---|---|---|---|---|---|---|
| | | | | Sham/ naïve | Sham + Rehab | Stroke | Stroke + Rehab | Total |
| | | | | 214 | 63 | 226 | 146 | 649 |
| Fig. 1 | a-f | Behavior | C57/BL6 | 9 | 8 | 10 | 9 | |
| Fig. 2 | a-i | Calcium imaging | C57/BL6 | 4 | NA | 7 | 6 | |
| Fig. 3 | d,e | Dendritic spine (stroke-projecting:L5) | C57/BL6 | 4 | 5 | 4 | 5 | Dendrite number: Basal; Sham= 19, Sham + Rehab=20, Stroke=27, Stroke+Rehab=22, Apical; Sham= 15, Sham + Rehab=25, Stroke=22, Stroke+Rehab=16 |
| | f-h | Rabies virus (stroke-projecting) | C57/BL6 | 9 | 8 | 9 | 11 | |
| Fig. 4 | a-f | Behavior + DREADD (stroke-projecting, acute) | C57/BL6 | 22 | NA | 25 | 27 | Sham/mCherry = 11, Sham/hM4D(Gi)=11, Stroke/mCherry=12, Stroke/hM4D(Gi)=13, Stroke+Rehab/mCherry=13, Stroke+Rehab/hM4D(Gi)=14 |
| Fig. 5 | a-e | Rabies virus (stroke-projecting) | C57/BL6 | 6 | 6 | 6 | 6 | |
| | h-l | GRASP | C57/BL6 | 7 | 9 | 7 | 9 | Neuron number: Sham= 24, Sham + Rehab=24, Stroke=21, Stroke+Rehab=22 |
| Fig. 6 | a-g | Behavior + DREADD (PV, chronic) | PV-Cre | 17 | NA | NA | 22 | Sham/mCherry= 9, Sham/hM4D(Gi)=8, Stroke+Rehab/mCherry=10, Stroke+Rehab/hM4D(Gi)=11 |
| Fig. 7 | a-k | Patch-clamp | C57/BL6 | 4 | NA | 3 | 4 | Neuron number: EPSC; Sham= 11, Stroke=12, Stroke+Rehab=13, IPSC; Sham= 12, Stroke=9, Stroke+Rehab=10 |
| Fig. 8 | a-j | Calcium imaging + DREADD (PV, acute) | PV-Cre | 6 | NA | 9 | 11 | Network number: Sham= 13, Stroke=17, Stroke+Rehab=19 |
| Fig. 9 | a-d | Mouse EEG | C57/BL6 | 4 | NA | 7 | 5 | |

| | | | | | | | | |
|---|---|---|---|---|---|---|---|---|
| Fig. 10 | b | IEG + drug | C57/BL6 | 15 | NA | NA | NA | Vehicle=5, AUT=5, DDL=5 |
| | c-d | Behavior + drug | C57/BL6 | 39 | NA | 35 | NA | Sham+Vehicle=12, Sham+AUT=13, Sham+DDL=14, Sham+Vehicle=11, Sham+AUT=12, Sham+DDL=12 |
| Supplementary Fig. 2 | a-c | Stroke model validation | C57/BL6 | NA | NA | 23 | NA | Normal stroke=9, cranial window=6, acrylic column=8 |
| Supplementary Fig. 3 | a-e | Bahavior + 2nd stroke | C57/BL6 | NA | NA | 14 | NA | Ipsi-RFA=7, Contra-CFA=7 |
| Supplementary Fig. 4 | c-d | Dendritic spine (corticospinal:L5) | C57/BL6 | 4 | 4 | 8 | 5 | Dendrite number: Basal; Sham= 23, Sham + Rehab=25, Stroke=26, Stroke+Rehab=26, Apical; Sham= 24, Sham + Rehab=25, Stroke=27, Stroke+Rehab=30 |
| | e-j | Rabies virus (corticocpinal) | C57/BL6 | 9 | 8 | 10 | 10 | |
| Supplementary Fig. 6 | a-b | Dendritic spine (L2-3) | C57/BL6 | 5 | 3 | 5 | 4 | Dendrite number: Basal; Sham= 18, Sham + Rehab=17, Stroke=20, Stroke+Rehab=22 |
| Supplementary Fig. 8 | b-c | Immunostaining (stroke-projecting) | C57/BL6 | 9 | NA | NA | NA | |
| Supplementary Fig. 9 | a-f | Immunostaining (interneurons) | C57/BL6 | 6 | 6 | 6 | 6 | |
| Supplementary Fig. 10 | a-c | GRASP validation | C57/BL6 | 4 | NA | NA | NA | |
| Supplementary Fig. 11 | a-j | IEG and perineurona nets | C57/BL6 | 6 | 6 | 6 | 6 | |
| Supplementary Fig. 12 | a-f | PV DREADD validation (PV, acute) | PV-Cre | 8 | NA | 9 | NA | |
| Supplementary Fig. 13 | a-g | Behavior + DREADD (stroke-projecting, chronic) | C57/BL6 | 17 | NA | 23 | NA | Sham/mCherry= 7, Sham/hM4D(Gi)=10, Stroke+Rehab/mCherry=11, Stroke+Rehab/hM4D(Gi)=12 |

**Supplementary table 3: The number of animals**
