# Supplementary material for: Parvalbumin interneurons regulate rehabilitation-induced functional recovery after stroke and identify a rehabilitation drug
Source: Nat Commun. 2025 Mar 15;16:2556. doi: 10.1038/s41467-025-57860-0 (PMC11910580; doi:10.1038/s41467-025-57860-0)
Supplement: Supplementary file 2 — Reporting Summary [file 41467_2025_57860_MOESM2_ESM.pdf]

nature portfolio

Corresponding author(s): Naohiko Okabe, S. Thomas Carmichael

Last updated by author(s): Mar 5, 2025

Reporting Summary

Nature Portfolio wishes to improve the reproducibility of the work that we publish. This form provides structure for consistency and transparency in reporting. For further information on Nature Portfolio policies, see our Editorial Policies and the Editorial Policy Checklist.

Statistics

For all statistical analyses, confirm that the following items are present in the figure legend, table legend, main text, or Methods section.

n/a	Confirmed	
☐	☒	The exact sample size (*n*) for each experimental group/condition, given as a discrete number and unit of measurement
☐	☒	A statement on whether measurements were taken from distinct samples or whether the same sample was measured repeatedly
☐	☒	The statistical test(s) used AND whether they are one- or two-sided<br>*Only common tests should be described solely by name; describe more complex techniques in the Methods section.*
☒	☐	A description of all covariates tested
☐	☒	A description of any assumptions or corrections, such as tests of normality and adjustment for multiple comparisons
☐	☒	A full description of the statistical parameters including central tendency (e.g. means) or other basic estimates (e.g. regression coefficient) AND variation (e.g. standard deviation) or associated estimates of uncertainty (e.g. confidence intervals)
☐	☒	For null hypothesis testing, the test statistic (e.g. *F*, *t*, *r*) with confidence intervals, effect sizes, degrees of freedom and *P* value noted<br>*Give P values as exact values whenever suitable.*
☒	☐	For Bayesian analysis, information on the choice of priors and Markov chain Monte Carlo settings
☒	☐	For hierarchical and complex designs, identification of the appropriate level for tests and full reporting of outcomes
☒	☐	Estimates of effect sizes (e.g. Cohen's *d*, Pearson's *r*), indicating how they were calculated

Our web collection on statistics for biologists contains articles on many of the points above.

Software and code

Policy information about availability of computer code

Data collection	NIS-Elements (Confocal), Scanbox (Calcium imaging), Igor 8.0 (mouse EEG), Net Station 4.5.3 software (human EEG)
Data analysis	Graphpad Prism 10 (Statistics), R 4.1.0 (graph and statistics), Imaris 10.0.1 (Image analysis), Matlab R2021a (Calcium imaging), EZcalcium (Calcium imaging), Igor 8.0 (mouse EEG), EEGLAB (human EEG)

For manuscripts utilizing custom algorithms or software that are central to the research but not yet described in published literature, software must be made available to editors and reviewers. We strongly encourage code deposition in a community repository (e.g. GitHub). See the Nature Portfolio guidelines for submitting code & software for further information.

Data

Policy information about availability of data

All manuscripts must include a data availability statement. This statement should provide the following information, where applicable:

- Accession codes, unique identifiers, or web links for publicly available datasets
- A description of any restrictions on data availability
- For clinical datasets or third party data, please ensure that the statement adheres to our policy

All data supporting the findings of this study are available within the paper and the Supplementary information

Research involving human participants, their data, or biological material

Policy information about studies with human participants or human data. See also policy information about sex, gender (identity/presentation), and sexual orientation and race, ethnicity and racism.

Reporting on sex and gender	20 Male, 7 Female stroke patients
Reporting on race, ethnicity, or other socially relevant groupings	19 White, 3 Asian control and 16 White, 8 Asian, 3 Black stroke patients<br>2 Hispanic, 25 Non Hispanic control and 2 Hispanic, 25 Non Hispanic stroke patients
Population characteristics	Age 49-67 years (median 58)<br>21 ischemic stroke patients and 6 intracerebral hemorrhage patients
Recruitment	Individuals with stroke aged 18 years or older were recruited from the inpatient rehabilitation facility at the University of California, Irvine Medical Center.
Ethics oversight	Approval was granted by the IRB at UC Irvine, where the reference number was 2004-3852. All the data from human participants were collected at University of California, Irvine, where the ethics approval was granted. All participants provided informed written consent

Note that full information on the approval of the study protocol must also be provided in the manuscript.

Field-specific reporting

Please select the one below that is the best fit for your research. If you are not sure, read the appropriate sections before making your selection.

[x] Life sciences [ ] Behavioural & social sciences [ ] Ecological, evolutionary & environmental sciences

For a reference copy of the document with all sections, see nature.com/documents/nr-reporting-summary-flat.pdf

Life sciences study design

All studies must disclose on these points even when the disclosure is negative.

Sample size	No sample size calculation was performed. Sample sizes were chosen based on previously published reports.
Data exclusions	All exclusion criteria were established prior to data collection: Mice showing poor motor behavior before stroke or no motor deficit after stroke were excluded prior to conducting analyses.
Replication	The exact number of repetitions are indicated in figures or figure legends. Experiments were successfully replicated with 2 to 3 cohorts of animals. Behavioral testing with AUT00201 and DDL-920 was conducted in a single animal cohort to ensure strict consistency between groups. All protocols used are described in detail in the Methods section.
Randomization	All samples used were randomly allocated across experimental conditions
Blinding	All procedures, including stroke surgery, behavioral tests, histological analysis, 2-photon calcium imaging analysis, and EEG analysis, were conducted by blinded experimenters, except for rehabilitation treatment.

Reporting for specific materials, systems and methods

We require information from authors about some types of materials, experimental systems and methods used in many studies. Here, indicate whether each material, system or method listed is relevant to your study. If you are not sure if a list item applies to your research, read the appropriate section before selecting a response.

Materials & experimental systems

n/a	Involved in the study	
[ ]	[x]	Antibodies
[ ]	[x]	Eukaryotic cell lines
[x]	[ ]	Palaeontology and archaeology
[ ]	[x]	Animals and other organisms
[x]	[ ]	Clinical data
[x]	[ ]	Dual use research of concern
[x]	[ ]	Plants

Methods

n/a	Involved in the study	
[x]	[ ]	ChIP-seq
[x]	[ ]	Flow cytometry
[x]	[ ]	MRI-based neuroimaging

Antibodies

Antibodies used	Rabbit anti-Parvalbumin (1:1000, Abcam, ab11427), Mouse anti-Parvalbumin (1:1000, Swant, 1275), Guinea Pig anti-Parvalbumin (1:1000, Synaptic system, 195 004), Rabbit anti-Somatostatin (1:1000, Peninsula lab, T-4103), Rabbit anti-Satb2 (1:1000, Abcam, ab92446), Rabbit anti-vGat (1:1000, Synaptic system, 131 003), Rabbit anti-FosB (1:1000, CST, 2251), Rabbit anti-Zif268 (1:1000, CST, 4154)
Validation	All the antibodies used in this study were commercially available and have been validated by the manufacturer

Eukaryotic cell lines

Policy information about cell lines and Sex and Gender in Research

Cell line source(s)	HEK293 cells (ATCC #CRL 3216)
Authentication	Cell line used was not authenticated. Cell line was used for AAV production.
Mycoplasma contamination	Cell line was not tested for mycoplasma contamination
Commonly misidentified lines (See ICLAC register)	No commonly misidentified cell lines were used

Animals and other research organisms

Policy information about studies involving animals; ARRIVE guidelines recommended for reporting animal research, and Sex and Gender in Research

Laboratory animals	2–4 month-old adult C57BL/6 (The Jackson Laboratory) or B6 PV-Cre (B6;129P2-Pvalbtm1(cre)Arbr/J, The Jackson Laboratory) male mice were used.
Wild animals	NA
Reporting on sex	All data were derived from male animals because female mice showed significantly higher mortality rates after stroke and poorer skill acquisition in skilled reaching behavior. Study with both sexes is warranted in future research.
Field-collected samples	NA
Ethics oversight	All procedures were performed under an NIH approved animal protocol and the University of California Los Angeles Chancellor's Animal Research Committee (ARC-2000-159-AM-011).

Note that full information on the approval of the study protocol must also be provided in the manuscript.

Plants

Seed stocks	NA
Novel plant genotypes	NA
Authentication	NA
